# Cancer Associated E17K Mutation Causes Rapid Conformational Drift in AKT1 Pleckstrin Homology (PH) Domain

**DOI:** 10.1371/journal.pone.0064364

**Published:** 2013-05-31

**Authors:** Ambuj Kumar, Rituraj Purohit

**Affiliations:** 1 Bioinformatics Division, School of Bio Sciences and Technology, Vellore Institute of Technology University, Vellore, Tamil Nadu, India; 2 Human Genetics Foundation, Torino, Italy; Institut Jacques Monod, France

## Abstract

**Background:**

AKT1 (v-akt murine thymoma viral oncogene homologue 1) kinase is one of the most frequently activated proliferated and survival pathway of cancer. Recently it has been shown that E17K mutation in the Pleckstrin Homology (PH) domain of AKT1 protein leads to cancer by amplifying the phosphorylation and membrane localization of protein. The mutant has shown resistance to AKT1/2 inhibitor VIII drug molecule. In this study we have demonstrated the detailed structural and molecular consequences associated with the activity regulation of mutant protein.

**Methods:**

The docking score exhibited significant loss in the interaction affinity to AKT1/2 inhibitor VIII drug molecule. Furthermore, the molecular dynamics simulation studies presented an evidence of rapid conformational drift observed in mutant structure.

**Results:**

There was no stability loss in mutant as compared to native structure and the major cation–π interactions were also shown to be retained. Moreover, the active residues involved in membrane localization of protein exhibited significant rise in NHbonds formation in mutant. The rise in NHbond formation in active residues accounts for the 4-fold increase in the membrane localization potential of protein.

**Conclusion:**

The overall result suggested that, although the mutation did not induce any stability loss in structure, the associated pathological consequences might have occurred due to the rapid conformational drifts observed in the mutant AKT1 PH domain.

**General Significance:**

The methodology implemented and the results obtained in this work will facilitate in determining the core molecular mechanisms of cancer-associated mutations and in designing their potential drug inhibitors.

## Introduction

AKT1 (v-akt murine thymoma viral oncogene homologue 1) kinase is a member of possibly the most frequently activated proliferation and survival pathway in cancer [1]–[6]. AKT gene family members are associated with cancer in human; especially prominent is the genetic amplification of AKT2. Point mutation in AKT1 has been detected in Proteus syndrome [2], endometrial carcinomas [4], [7] and leukaemia [5], [8]–[9], gene amplification in a single gastric carcinoma out of a screen of more than 225 diverse human malignancies [10], and in 1 gliosarcoma out of 103 malignant glial cancers [11]. In another study it was found that AKT1 kinase activity was frequently elevated in several high-grade, late-stage cancers [12] although the exact mechanisms of such observations are still unclear. The activation of AKT1 is driven by membrane localization, which is in turn initiated by the binding of the pleckstrin homology (PH) domain to phosphatidylinositol-3,4,5-trisphosphate (PtdIns(3,4,5)P3) or phosphatidylinositol-3,4-bisphosphate (PtdIns(3,4)P2), followed by phosphorylation of the regulatory amino acids serine 473 (Ser 473) and threonine 308 (Thr 308) [3], [13]. The pathological association of AKT with the plasma membrane is a common thread that connects AKT to cancer [1]–[7], [14]–[15]. The oncogenic behaviour of the Gag-AKT fusion protein from the murine leukaemia retrovirus AKT8 (v-akt murine thymoma viral oncogene homologue 8) requires a membrane-targeting myristoylation signal–the first presumptive evidence that pathological membrane localization of AKT1 kinase activity could be transforming in mice [16]. Moreover, the importance of AKT in human cancer is largely inferred from frequently occurring mutations in the enzymes that regulates the activity of these second messenger phospholipids (PtdIns(3,4,5)P3, PtdIns(3,4)P2) and ultimately causes the activation of AKT through membrane recruitment [1], [3], [17], [18]. Tumours samples from the patients with breast, colorectal cancer and cases of leukaemia has been shown to frequently harbour activating somatic mutations in AKT1 [1], [5]. In addition, the loss of associated interacting members such as the phosphatase and tensin homologue (PTEN) lipid phosphatase activity has been reported in glioblastoma, prostate and endometrial cancers by means of somatic mutations [11], or in breast cancer by means of epigenetic silencing [12], which represents an alternative indirect mechanism for activating AKT. Hence, AKT1 seems to have a crucial but passive role in oncogenesis and acts as an indirect intermediary between mutated upstream regulatory proteins and downstream signalling molecules.

The oncogenic activation of AKT1 can be induced by several means, most commonly occurring either due to the compromise in its membrane-targeting by PH domain, or due to the pathological conformational changes occurring in the mutant structure. The genetic mutations in PH domain has been previously reported to disturb the localization behaviour and in loss of sensitivity towards the PtdIns and has led to major consequences to its functional behaviour [1]. While screening the causes behind such observation, the computational approach forms a significant backbone and serves in carrying keen experimental observations in low cost input. A point mutation at nucleotide 49 that results in a lysine substitution for glutamic acid at amino acid 17 (AKT(E17K)) has been implicated in cancer cases [1], [3], [19]–[21]. The molecular cause behind the associated oncological outcome has not yet been studied in much detail. It is known that the binding of phosphoinositides to the PH domain activates AKT1. In the apo conformation, Glu 17 occupies the phosphoinositide-binding pocket and forms a network of hydrogen bonds. In the work of Carpten et al. (2007), it was shown that the Lys 17 substitution results in a shift in the surface charge around the pocket from negative with Glu 17 to effectively neutral in the mutant [1]. This mutation has resulted in an increased level of AKT phosphorylation on Thr 308 and Ser 473 as compared to wild-type. AKT1 (E17K) kinase activity was shown to be approximately four fold higher than that of AKT1 (WT), suggesting that the mutation has altered the AKT1 regulation and hence it enhances cellular activity [1]. Furthermore, it has also been proposed that the mutation induces large affinity increase for PI(4,5)P2 which is essential to the constitutive plasma membrane targeting of the mutant PH domain and thus to the oncogenic nature of the full-length E17K AKT1 protein [3]. Moreover, it was also suggested that the E17K PH domain mutation caused structural changes in the PH domain, which further hindered its interaction with AKT1/2 inhibitor VIII [1]. All these observations strongly suggest that the damaging conformational changes in mutant PH domain might have caused such pathological outcomes.

The Molecular dynamic simulation (MDS) has been a promising approach to investigate the conformational changes in the mutant protein structure with respect to its native conformation [22]–[29]. We know that change in the conformational orientation of a protein molecule affects its interaction with ligand as well as its biological partners. In this work we focussed our study on investigating the changes in dynamic behaviour of AKT1 PH domain induced by the cancer causing E17K mutation. Previous investigations have shown that E17K mutation has a potential role in inducing significant alteration in AKT1–PH domain conformation as well as in inducing resistance against AKT1/2 inhibitor VIII [1]. We conducted MDS in notion to infer the conformational changes occurring in the mutant structure which may account for the observed molecular changes and the associated pathological outcomes. Moreover, the docking studies also assisted in determining the changes in the ligand interaction affinity of protein. Overall, our results provided a strong evidence of major conformational drift occuring in mutant protein as compared to the native.

## Materials and Methods

### Datasets

We selected crystal structures of native PH (PDB ID: 1UNP), E17K structure (PDB ID: 2UZS) and structure of AKT1/2 inhibitor VIII (PDB ID: 3O96) from Brookhaven Protein Data Bank [30] for our investigation. The obtained structures were energy minimized using GROMOS96 43a1 forcefield through gromacs 4.5.4.package [31].

### Cation–π Interactions

Cation–π interactions were computed using CaPTURE program [32]. Cation–π interactions in protein structures were identified and evaluated by using an energy-based criterion for selecting significant sidechain pairs. The percentage composition of a specific amino acid residue contributing to cation–π interactions is obtained from the equation:
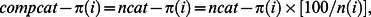
where “i” stands for the five residues (Lys, Arg, Phe, Trp, and Tyr), *n*cat–π is the number of residues involved in cation–π interactions, and *n*(i) is the number of residues of type “i” in the considered protein structures.

Furthere the cation-π interaction is increasingly recognized as an important noncovalent binding interaction relevant to structural biology. It uses a variant of the optimized potentials for liquid simulations (OPLS) force field to provide an energetic evaluation of all potential cation-π interactions in a protein. The electrostatic energy (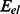) is calculated using the equation:
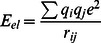
where 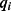 and 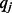 are the charges for the atoms *i* and *j*, respectively, and 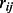 is the distance between them. The van der Waals energy is given by:



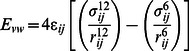



Where 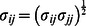 and 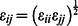; 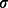 and 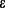 are the van der Waals radius and well depth, respectively.

If 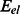
≤−2.0 kcal/mol, the pair is counted as a cation-π interaction. If 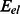
>−1.0 kcal/mol, the structure is rejected. If −2.0<
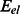
≤−1.0 kcal/mol, the structure is retained only if 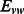
≤−1.0 kcal/mol.

### Ligand Binding Cavity Analysis

Ligand binding cavity of AKT1 PH domain structure was examined by CASTp tool [33]. CASTp implements incremental regular triangulations, commonly referred as weighted Delaunay triangulation and the alpha complex to measure the shape complimentarity of given macromolecule. It also measures the surface accessible pockets and the interior cavities that are inaccessible. Measures follow the analytical calculations identifying area and volume of each pocket and cavity [33].

### Complementarity Analysis

We used the web server PatchDock [34] to compute the complementarity of AKT1/2 inhibitor VIII and AKT1 PH domain. The underlying principle of this server is based on molecular shape representation, surface patch matching plus filtering and scoring. It is aimed at finding docking transformations that yield good molecular shape complementarity. Such transformations, when applied, induce both wide interface areas and small amounts of steric clashes. Segmentation algorithm is implementated for detection of geometric patches. The patches are filtered, so that only patches with ‘hot spot’ residues are retained. Further, hybrid of the Geometric Hashing and Pose-Clustering matching techniques are implimented to match the patches. All complexes with unacceptable penetrations of the atoms of the receptor to the atoms of the ligand are discarded. Finally, the remaining candidates are ranked according to a geometric shape complementarity score. 3D coordinates of native and mutant structures were subjected to docking with AKT1/2 inhibitor VIII.

### Docking Analysis

Molecular docking studies were performed to investigate the role of mutation in affecting the AKT1/2 inhibitor VIII binding activity of kinesin domain using Autodock 4.0 [35]. AutoDockTools 1.4.6 was used for establishing the Autogrid points as well as visualization of docked ligand-amino acid structures [35]. Grid map centred on the ligands binding sites of native and mutant structures were constructed to cover the AKT1/2 inhibitor VIII binding pockets. Lamarckian genetic algorithm was used to carry out molecular docking simulations. Simulations were performed using up to 2.5 million energy evaluations with a maximum of 27000 generations. The lowest energy conformations were regarded as the binding conformations between AKT1/2 inhibitor VIII and PH domain.

### Molecular Dynamics Simulation

Molecular Dynamics Simulation was performed by using gromacs 4.5.4 package [31]. Structures of native and mutant PH were used as starting point for MD simulations. Systems were solvated in a rectangular box with TIP3P water molecules at 10 Å marginal radius. At physiological pH the structures were found to be negatively charged, thus in order to make the simulation system electrically neutral, we added 2 chloride ions (CL^−^) in the simulation box using the “genion” tool that accompanies with gromacs package. Initially the solvent molecules were relaxed while all the solute atoms were harmonically restrained to their original positions with a force constant of 100 kcal/mol for 5000 steps. Emtol convergence criterion of 1000 kcal/mol and fourierspacing of 0.12 nm was used. After this, whole molecular system was subjected to energy minimization by using conjugated gradient method. Berendsen temperature coupling method [36] was used to regulate the temperature inside the box. Isotropic pressure coupling was performed using Parrinello-Rahman method. Electrostatic interactions were computed using the Particle Mesh Ewald method [37]. The ionization states of the residues were set appropriate to pH 7 with all histidines assumed neutral. The pressure was maintained at 1 atm with the allowed compressibility range of 4.5e^−5^ atm. SHAKE algorithm was used to constrain bond lengths involving hydrogen, permitting a time step of 2 fs. Van der Waals and coulomb interactions were truncated at 1.0 nm. The non-bonded pair list was updated every 10 steps and conformations were stored every 0.5 ps. Position restraint simulation for 2000 ps was implemented to allow solvent molecules to enter the cavity region of structure. Finally, systems were subjected to MD simulation for 50 ns. We computed the comparative analysis of structural deviations in native and mutant PH structure. RMSD, RMSF, SAS and Rg analysis were carried out by using g_rms, g_rmsf, g_sas and g_gyrate tool respectively. Number of distinct hydrogen bonds formed by specific residues to other amino acids within the protein during the simulation (NHbond) was calculated using g_hbond. NHbond determined on the basis of donor–acceptor distance smaller than 0.35 nm and of donor–hydrogen-acceptor. Graphs were plotted using Grace GUI toolkit 5.1.22 version.

## Results and Discussion

Carpten et al. (2007) proposed that the E17K mutation had caused major elevation in AKT1 activity [1]. According to the inferences presented in their work, the mutation causes significant change in the conformation of AKT1 PH domain which ultimately leads to over phosphorylation, 4.5 fold rise in its membrane localization tendency and elevation of kinase activity which can lead to cancer [1]–[6]. Moreover, the mutation causes loss in binding with AKT1/2 inhibitor VIII. Although the experimental outcomes have presented the marginal overview of the molecular mechanism associated with E17K mutation, we still lack the detailed explanation of the observed phenotype at molecular and atomic level. In order to understand the associated structural and molecular changes, we conducted molecular docking and molecular dynamics simulation. Results obtained in our study provided interesting inferences, pointing towards rapid conformational drift in the E17K PH domain. First we examined the most accessible ligand binding cavity of AKT1 PH domain using CASTp tool. From the CASTp results, it was clear that the residues 38 to 52 and 77 to 87 amino acid positions forms the most accessible cavity regions for ligand interaction. To examine the change in binding affinity in native and mutant protein, we further conducted complimentarity analysis using patchdock tool. In patchdock results the total docking score of mutant with ligand were found to be lower as compared to native structure (Table 1).

**Table 1:** PatchDock’s Docking score, ligand–receptor interaction energy of native and mutant AKT1PH domain with ATP.

Structure	Docking Score	Ligand-Receptor Electrostatic Energy (in Kcal/mol)	Ligand-Receptor Van der Waals Energy (in Kcal/mol)	Total Ligand-Receptor Interaction Energy (in Kcal/mol)
Mutant	4694	−0.11	−2.45	−3.87
Native	5326	−0.43	−9.39	−12.32

**Figure 1:**
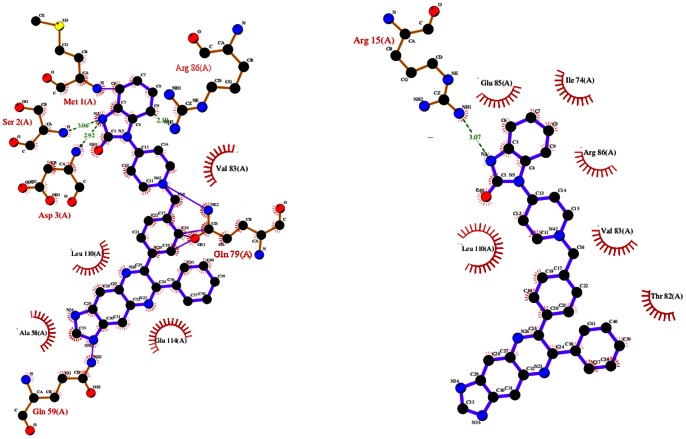
Interaction between native/mutant AKT1 PH domain and AKT1/2 inhibitor VIII. Structure on the left shows native PH- AKT1/2 inhibitor VIII complex and structure on the right shows mutant PH (E17K) - AKT1/2 inhibitor VIII complex.

Molecular docking analysis was conducted using autodock 4.0 package to unravel the changes in AKT1/2 inhibitor VIII binding affinity in mutant structure as compared to native. A notable loss of interaction affinity was observed in mutant structure as compared to native. In native the optimal binding energy was −12.32 kcal/mol whereas in mutant it was found to be −3.87 kcal/mol (Table 1). A better insight of change in molecular interaction can be seen in Fig. 1. A total of 6 amino acid residue interactions with AKT1/2 inhibitor VIII were observed in the native structure, whereas in mutant there was only one interaction found. Moreover, the patterns of native interactions were not retained in the mutant structure. The residues Met 1, Ser 2, Asp 3, Gln 59, Gln 79 and Arg 86, which were actively participating in inhibitor interaction in native, did not show any role in mutant structure (Fig. 1). These molecular interactions revealed the damaging consequences of mutation on AKT1/2 inhibitor VIII interaction affinity of PH domain.

**Figure 2:**
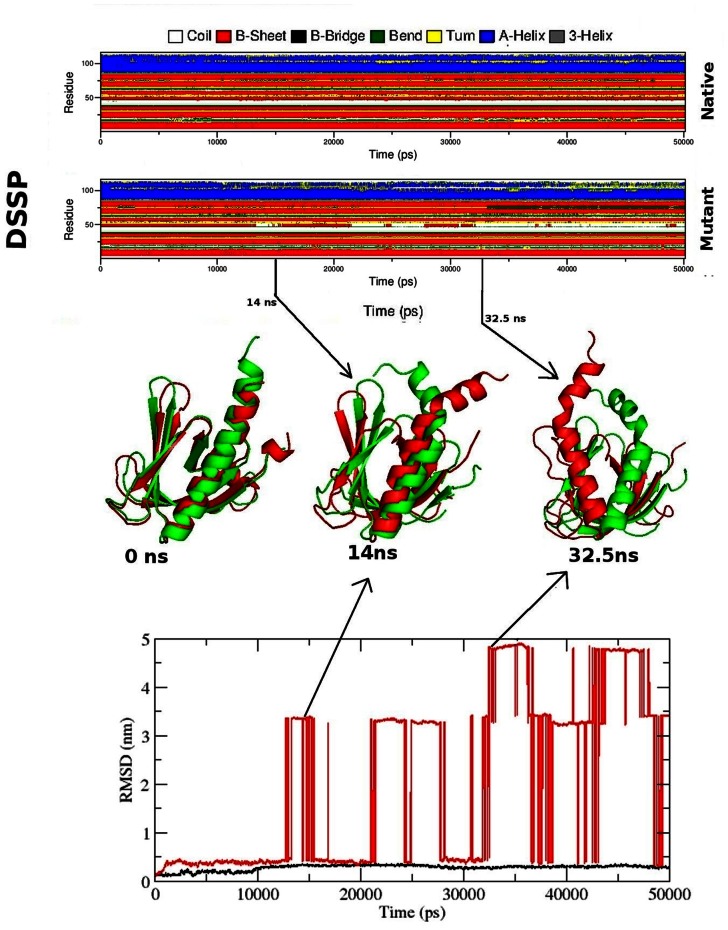
RMSD and DSSP changes in native and mutant structure throughout the simulation. Figure shown at the top represents native and mutant DSSP plot. In the middle, the superimposed native and mutant structures are shown. Here the native is shown in green and mutant in red. At the bottom, the RMSD plot is shown. Native is shown in black and mutant in red.

**Figure 3:**
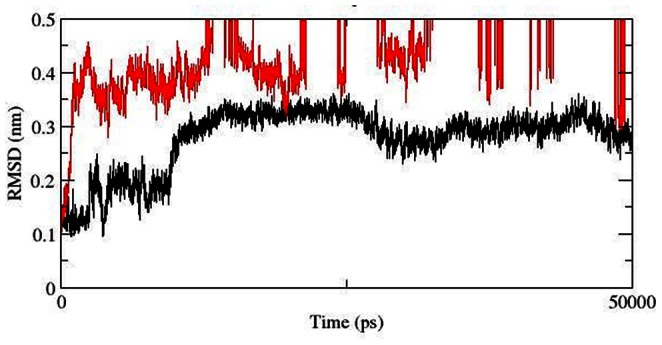
In order to show the deviations clearly, the RMSD plot is shown for upper RMSD limit of 0.5 nm. Native is shown in black and mutant in red.

**Figure 4:**
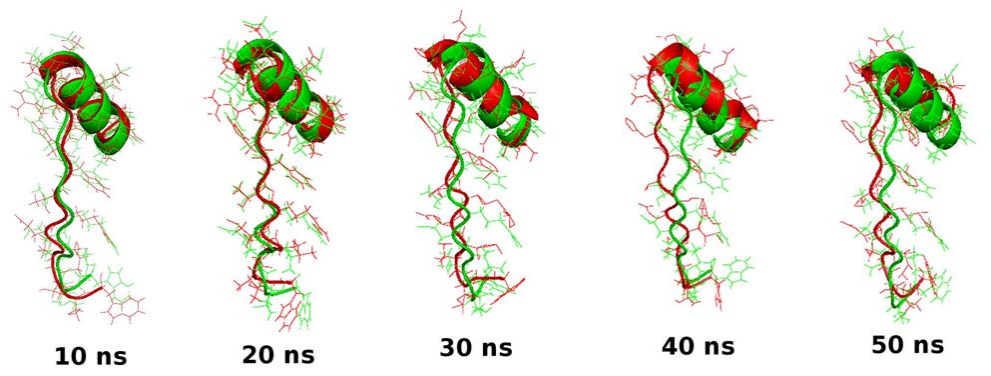
Superimposed native and mutant structures of residue region 80–105 at different time steps. Here the native is shown in green and mutant in red.

Protein-ligand interactions were often accompanied by significant changes in their conformation. Thus to investigate the root cause of AKT1/2 inhibitor VIII binding affinity loss in mutant structure and to understand the structural consequences of E17K mutation, we conducted molecular dynamics simulation of native and the mutant AKT1 PH domain. We investigated RMSD, RMSF, Rg, SASA and NHbond variation, and distance fluctuations between the important interacting residues in the native and mutant structure. RMSD for all the Cα atoms from the initial structure were calculated which were considered as the central origin to measure the protein system. Here the RMSD measures average distance between the atoms of protein at the two consecutive states. In Fig. 2 and Fig. 3, native and mutant PH protein showed distinct fashion of deviation till 13 ns from their starting structure, resulting in backbone RMSD ∼0.3 in native and ∼0.4 in mutant. After 13 ns, mutant PH protein showed extreme deviation pattern till the end of the simulation when compared to native. At 13137 ps the mutant structure showed an abrupt rise in RMSD value reaching up to 3.36 nm and further returned to 0.44 nm at 16078 ps (Fig. 2, Fig. 3). This trend of fluctuation continued in the mutant structure till the end and showed extreme deviations when compared to native. Moreover the mutant reached up to its highest RMSD level at 35931 ps, reaching up to 4.9 nm. All these data’s suggested that the mutant structure had undergone distinct conformational drift throughout the simulation, whereas such drifts were not observed in the native structure. The above observed conformational drifts were further in concordance to the results obtained by the analysis of time-dependent secondary structure fluctuations through DSSP analysis. The mutant structure terminal residues showed conformational drift from alpha-Helix to bend form, while such drifting was not seen in native structure (Fig. 2). Furthermore, extreme conformational drifts were observed in mutant between 25 ns–35 ns. The amount of bends considerably increased after 34 ns timestep in mutant structure (Fig. 2). Particularly in the residue region 80 to 105, distinct fashion of conformational drift from alpha helix to bend and coil form were observed in the mutant structure (Fig. 2), and is shown in Fig. 4. All results were closely in concordance to the RMSD deviations (Fig. 2, Fig. 3, Fig. 4). We have also showed the superimposed structures for native and mutant at the beginning of the simulation and for specific time steps where the conformational drifts occurred at higher range (Fig. 2). It seemed as if the rapid drifting had also caused certain domain transitions and conformational changes in the mutant structure, specially in the C terminal region of the PH domain, as compared to native.

**Figure 5:**
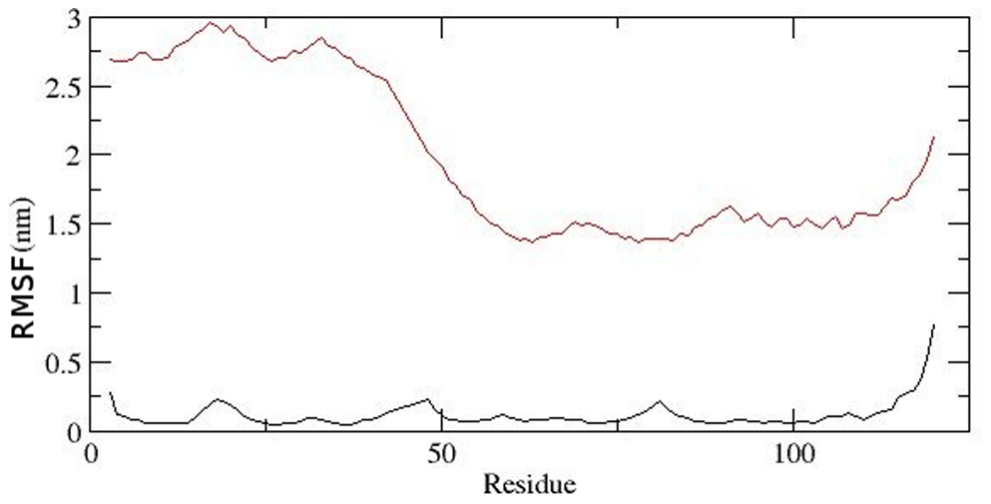
RMSF of the backbone CAs of Cα atoms of native and mutant AKT1 protein PH domain versus time at 300 K. Native is shown in black and mutant in red.

**Figure 6:**
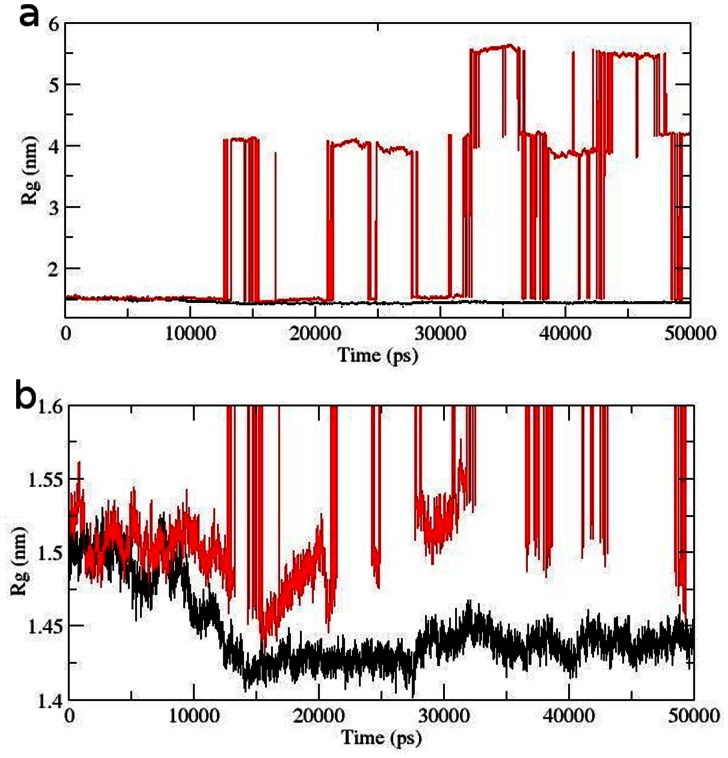
Radius of gyration of Cα atoms of native and mutant AKT1 protein PH domain versus time at 300 K. (a) Complete Rg fluctuations. (b) restricted to maximum upper Rg limit of 6 nm. Native is shown in black and mutant in red.

With the aim of determining whether mutation affected the dynamic behaviour of residues and to examine the cause of such conformational drifts observed in RMSD and DSSP results, the RMSF values of native and mutant backbone residues were calculated (Fig. 5). RMSF values of mutant residues were found to be significantly higher in mutant structure as compared to native (Fig. 5). This showed that the observed drifting was accompanied by increase in atomic fluctuations in the mutant structure. The radius of gyration (Rg) is defined as the mass-weight root mean square distance of collection of atoms from their common centre of mass. Hence it gives an insight into the overall dimension of protein and has assisted in examining the notion of rapid conformational drifts observed in the mutant structure. Radius of gyration plot for Cα atoms of protein vs time at 300 K is shown in Fig. 6. The Rg fluctuations in mutant structure were very similar to the above observed RMSD changes. After 13137 ps an abrupt rise in Rg values in mutant structure were observed, reaching up to its highest level of 4.1 nm, whereas the Rg value of native structure remained at 1.5 nm. Moreover, at 35931 ps it reached up to the maximum value of 5.62 in mutant. At the end of simulation, the mutant structure showed Rg value of 4.16 whereas in native it was found to be 1.42.

**Figure 7:**
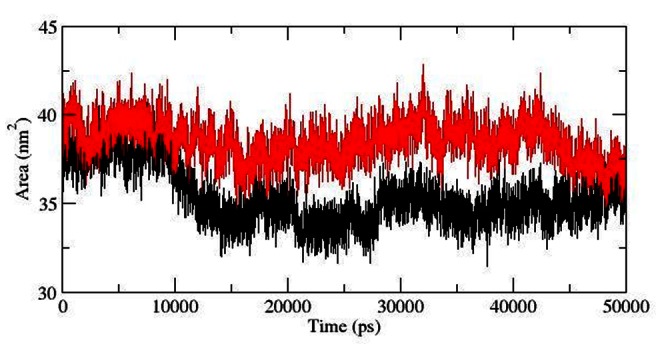
Solvent-accessible surface area (SASA) of native and mutant AKT1 protein PH domain versus time at 300 K. Native is shown in black and mutant in red.

**Figure 8:**
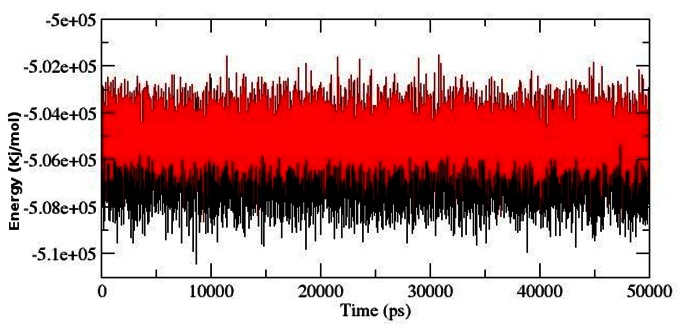
Total energy fluctuation in native and mutant AKT1 protein PH domain versus time at 300 K. Native is shown in black and mutant in red.

Solvent accessibility surface area accounts for bimolecular surface area assessable to solvent molecules. Rise in SASA value in mutant structure denotes it’s relative expansion as compared to native. The change of SASA of native and mutant protein with time is shown in Fig. 7. Mutant protein indicated greater value of SASA with time, while native showed lower SASA value. Longer fluctuation in Rg plot indicated that the mutant protein as well as the ATP binding domain might be undergoing a significant structural transition. This was further supported by SASA result where the mutant was found to exhibit large SASA as compared to native. All these observation collectively suggested that the mutant might have undergone drifting which ultimately led to its transition into an open conformation and might have occurred due to the stability losses in protein structure. Thus to elucidate if the mutation had caused any damages to the structural stability of protein during the process of drifting, we investigated the total energy fluctuation throughout the simulation. The results showed very slight rise in total energy value in mutant structure which ruled out the notion of stability losses induced by mutation (Fig. 8).

**Figure 9:**
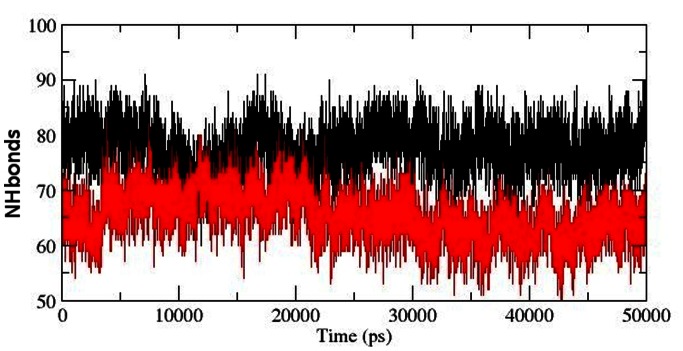
Average number of protein–solvent intermolecular hydrogen bonds in native and mutant MCAK protein motor domain versus time at 300 K. Native is shown in black and mutant in red.

**Figure 10:**
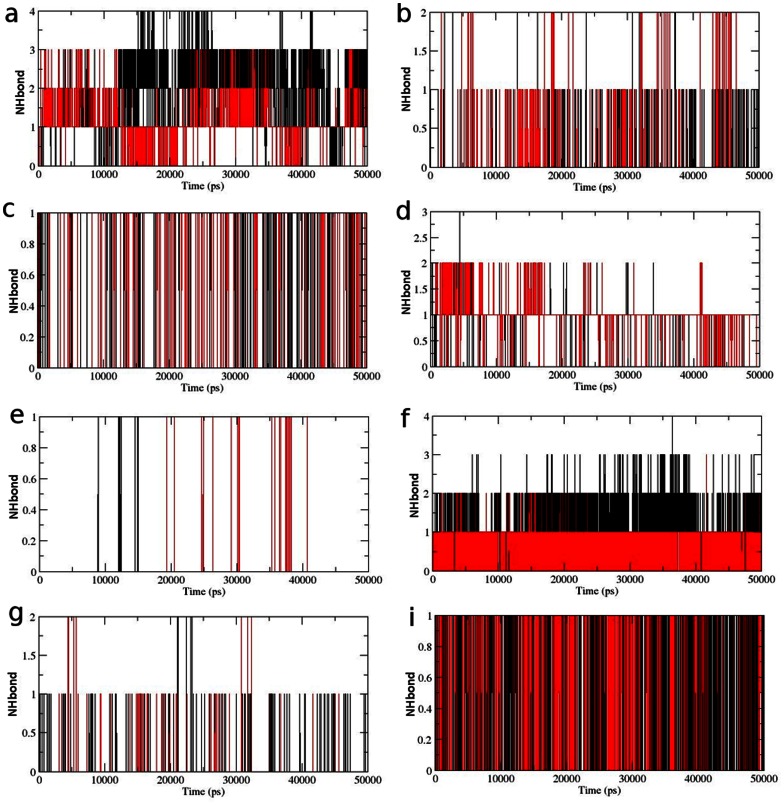
Average number of protein–solvent intermolecular hydrogen bonds in native and mutant AKT1 protein PH domain membrane localization residues versus time at 300 K. (a) K30 (b) K32 (c) W36 (d) R38 (e) R40 (f) Q53 (g) R56 (h) K57 (i) V58. Native is shown in black and mutant in red.

Hydrogen bond is the major factor for maintaining the native conformation of protein and protein flexibility is directly proportional to intramolecular NHbonds between amino acid residues [38]–[40]. Intramolecular NHbond analysis of native and mutant proteins were performed with respect to time in order to understand the relationship between flexibility and hydrogen bond formation. Mutant structure showed a significantly less number of NHbond formation during the simulation when compared to native structure (Fig. 9). This observation was in concordance to the rise in Rg and SASA values in mutant and strongly suggested that the observed conformational drifts and increase in atomic fluctuations might have aroused due to the loss of NHbond formation in protein. Moreover, the amino acid residues K30, K32, W36, R38, R40, Q53, R56, K57 and V58 had been shown to be active components in their localization to membrane [41]. Thus we calculated the number of NHbond forming by these residues to study the effect of mutation on membrane localization. Although the total number of NHbonds formed in the mutant structure were significantly lower as compared to native, the total number of NHbonds formed in these residues did not suffer from any major losses imposed by mutation. Loss in NHbond formation was observed in residues K30 and Q53, whereas other residues has either showed higher number of NHbonds or maintained the native count of NHbonds throughout the simulation in the mutant structure (Fig. 10). These results were also in strong concordance to the observation reported by Carpten et al., (2007) [1] where it was shown that the mutation had caused significant rise in membrane localization potential of protein. The rise in NHbond formation in these residues could be a strong factor in explaining the rise in membrane localization potential of mutant protein.

**Figure 11:**
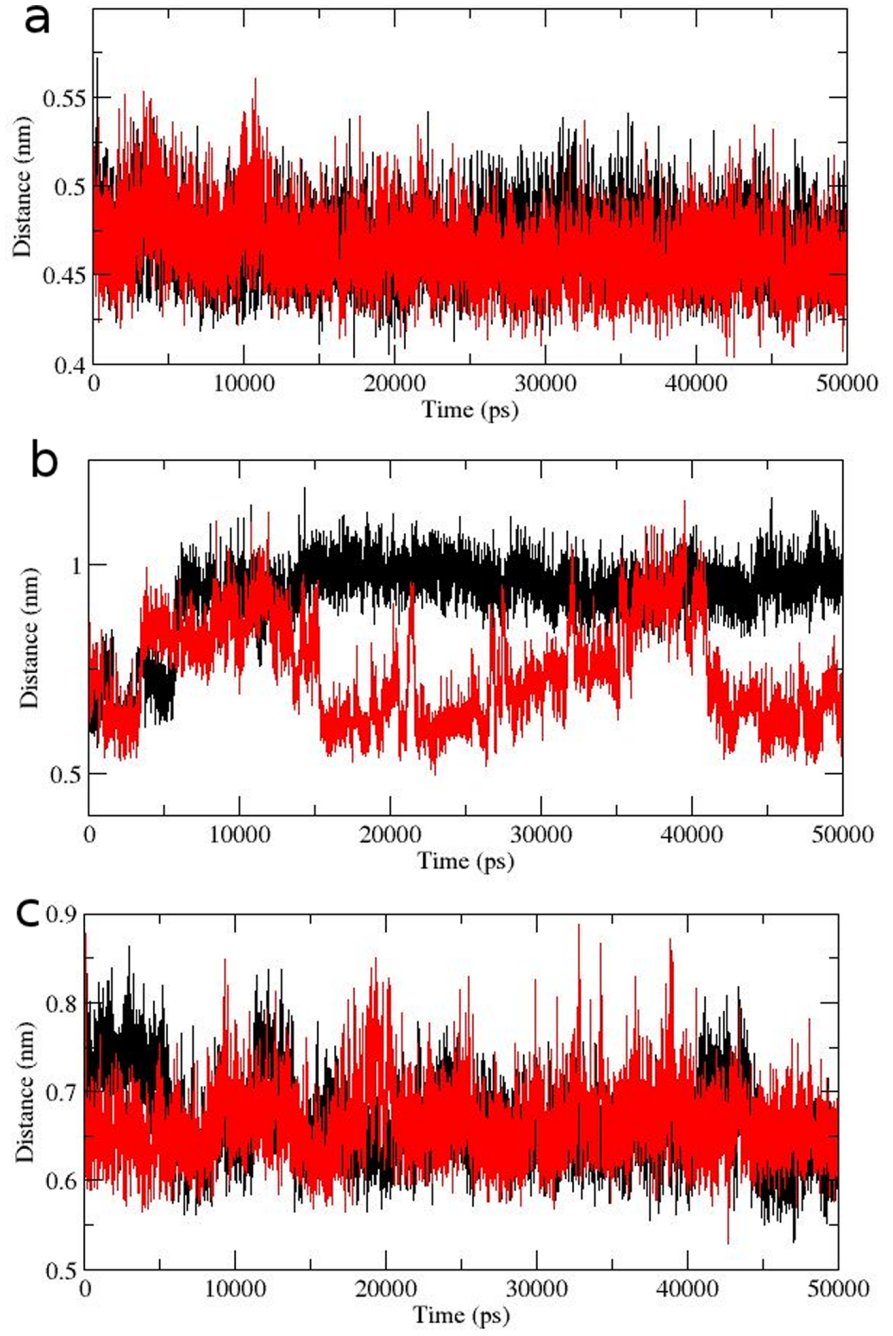
Distance fluctuation plot for native and mutant Cation-π interaction residues. **(a)** Arg86-Phe55 **(b)** Lys8-Trp99 **(c)** Arg69-Trp22. Native is shown in black and mutant in red.

The importance of cation–π interaction had been examined in several researches for their corresponding role in maintaining the stability of proteins. We investigated a total of 3 energetically significant cation–π interactions including Arg86-Phe55 (Fig. 11a), Lys8-Trp99 (Fig. 11b) and Arg69-Trp22 in the AKT1 PH domain (Fig. 11c). To examine if the cation–π interactions are retained in mutant structure, we further investigated the bond length fluctuations for these distant cation–π interaction. It was notable observation that the cation–π interactions in native as well as mutant were retained. Moreover, the Arg69-Trp22 interaction showed distinct fluctuation pattern and might be playing influential role in inducing conformational drift (Fig. 11c). By collectively studying all the structural and molecular changes in the AKT1 PH domain we could say that the observed conformational drifting, rise in atomic fluctuations and loss of total NHbonds in the mutant structure significantly contributed in causing observed pathological consequences imposed by E17K mutation. Furthermore, the 4 fold rise in membrane localization potential might have been induced by rise in NHbonds in active localization residues of the PH domain. These observations led us to the conclusion that the cancer associated phenotype observed in case of AKT1 PH domain E17K mutation is caused by the reported molecular and conformational changes which also cause resistance to the AKT1/2 inhibitor VIII by inducing shape drifting in its corresponding binding pocket.

### Conclusion

Amino acid variants usually induces pathological outcomes by damaging the native conformation of protein. The E17K mutation in AKT1 PH domain had been reported to induce cancer and resistance to AKT1/2 inhibitor VIII. To examine the detailed molecular mechanism behind such outcome, we conducted molecular docking and molecular dynamics simulation of native and mutant structure. The stability and important amino acid interactions were retained in the mutant structure, which ruled out the possibility of damaging effect of mutation. Furthermore, an abrupt rise in RMSD values and conformational drifts were observed in mutant sructure. Moreover, the resistance to AKT1/2 inhibitor VIII might had been caused by the changes in conformational structure of the binding pocket. In conclusion, the overall result exaplained that the molecular cause of 4 fold rise in mutant protein localization and kinase activity, which has ultimately caused cancer associated phenotypes.
